# African nurses on the move: decisions, destinations and recruitment practices - a scoping review

**DOI:** 10.1186/s12913-025-12531-0

**Published:** 2025-03-22

**Authors:** Fuseini Adam, Sioban Nelson, Bukola O. Salami, Quinn Grundy, Osman Wahab

**Affiliations:** 1 Lawrence Bloomberg Faculty of Nursing, University of Toronto, Toronto, ON Canada; 2 Department of Community Medicine, University of Calgary, Calgary, AB Canada; 3 Department of Medicine, Northern Regional Hospital, Tamale, Ghana; 4 Department of Adult Health, School of Nursing and Midwifery, University for Development Studies, Dungu, Tamale, Ghana

**Keywords:** Nurse, Migration, Africa, Health human resources, Sustainable Development Goals, Universal health coverage, Nursing shortage

## Abstract

**Background:**

The transnational migration of African nurses negatively impacts nurse-to-population ratios and life expectancy indices in many African countries. Understanding migration decisions, destination preferences, and recruitment practices of African nurses is crucial for identifying appropriate and effective retention interventions.

**Objective:**

The objectives of this scoping review are to examine the state of evidence in relation to the decisions surrounding international African nurse migration, as well as destinations preferences and recruitment practices employed to attract African nurses.

**Methods:**

Guided by the updated Joanna Briggs Institute (JBI) methodology for scoping reviews, we conducted a comprehensive search on empirical studies and grey literature on African nurse migration published in English from 2000 onwards and indexed in health and interdisciplinary databases. Studies on African nurse or student nurse migration intention were excluded.

**Results:**

We included 28 studies, twenty-one of which were peer-reviewed and seven from the grey literature. Synthesis of included studies found that international African nurse migration is influenced by economic challenges and income disparities, and career dynamics and job sustainability in home countries. The choice of destination by African nurses is impacted by African countries' past colonial relationships with destination countries, linguistic and cultural similarities. African nurses are recruited through international inter-agency collaboration and via direct recruitment by destination country health systems.

**Conclusion:**

Low income, poor economic growth and inadequate investment in African health systems significantly drive African nurse emigration, complicating efforts to attain universal health coverage. Recruitment strategies for nurse from African are often unregulated and can lead to exploitation and human trafficking. Again, as African nurse migration continues to rise, further studies are needed to examine their migration and transition experiences, as well as the support systems available in their destinations. Finally, improving workforce policies to meet the evolving needs of nurses is vital for retaining nurses in Africa.

## Background

The quality and efficiency of healthcare delivery depend on a well-trained and optimally supplied nursing workforce [1]. Nurses constitute the cornerstone of health delivery systems – they possess a complex blend of invaluable skills and an expansive scope of practice which are crucial for effective health provision [2]. Consequently, the availability of nurses directly impacts the ability of health delivery systems to provide timely care [2]. To achieve optimal population health and meet targets such as Universal Health Coverage (UHC) and the Sustainable Development Goals (SDGs), African health systems must maintain an adequate supply of nurses. Out of the total population of 3.6 million African health workers across 47 countries, 37 percent (1,332,000) are nurses and midwives [3]. Africa’s nurse-to-population ratio averages 18 per 10,000, falling below the global threshold of 29 [4], which indicates a nursing shortage.

**Fig. 1:**
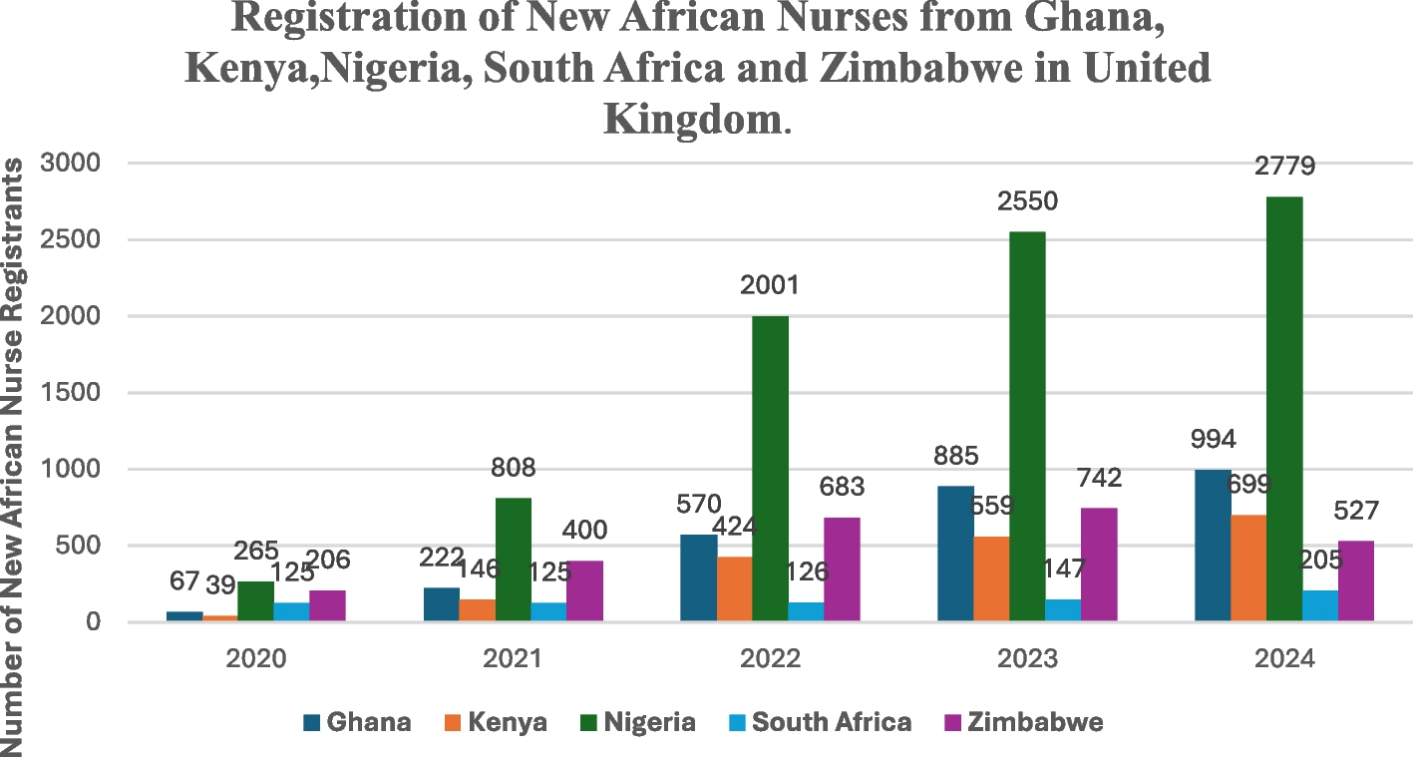
Annual registration of new Ghanaian, Kenyan, Nigerian, South African and Zimbabwean nurses in the United Kingdom. Source: NMC-UK (2024)

One of the factors exacerbating the nursing shortage in Africa is migration [5]. Labour migration is defined as “the voluntary movement of workers from one employment station to another in search of different working arrangements” [6]. Due to considerable variability and a lack of disaggregated data on the migration of African nurses, it is challenging to accurately gauge statistics on the trend of African nurse migration [6]. Despite these limitations, studies have documented a steady rise in the migration of nurses from countries in Africa [6, 7]. Their emigration imposes substantial economic costs, depletes nursing workforces, undermines health care delivery and lowers the morale of the remaining workforce on the continent [3, 8–11]. As illustrated in Fig. 1, the United Kingdom’s new permanent registration data for overseas nurses, suggest an increase in registrations from the top five African source countries, including Nigeria, Kenya, Ghana, South Africa and Zimbabwe between 2021 and 2024. Nigerian nurses experienced the most significant growth, with registrations increasing from 808 in 2021 to 2,779 in 2024, reflecting a 343.9% increase.

The World Health Organization (2020) reports that Africa does not have a nursing workforce proportionate to the targets for achieving UHC and the Sustainable Development Goals (SDGs). For instance, the recommended nurse-to-population ratio for meeting UHC target is 83 nurses per 10,000 or 8.3 nurses per 1,000 population, however, Sub-Saharan Africa has fewer than 20 nurses per 10,000 or 2.0 nurses per 1,000 population. This reflects the widening gap in nursing density variation in a continent with a population of over one billion but only 3 precent of global health workforce [12]. The exigent need to improve nurse-population indices could not be more pressing. Declining nurse-to-population statistics are particularly concerning because governments struggle to enough employ nurses to fill vacant positions, especially in rural areas, leading to maldistribution of nurses [3, 5, 13]. Health outcomes deteriorate when health workers, including nurses emigrate from low- and middle-income countries (LMICs), including those in Africa [13, 14]. The distressed state of the nursing workforce has been a significant factor in the decline in life expectancy in several Sub-Saharan African (SSA) countries [5]. For instance, a cross-sectional study on the relationship between nursing workforce and life expectancy found that, globally and regionally, the nursing workforce is a significant independent contributor to life expectancy at birth [15, 16]. This demonstrates a clear relationship between the availability of a nursing workforce, improved health outcomes and life expectancy.

*The World Migration Report* by the International Organization for Migration [17, 18] highlights the complexity of international migration and reiterated that globalization has increased human interconnectedness across multiple aspects of social, economic, and security dimensions of human endeavors [18]. The report argues that migration is linked to a range of important factors including, geopolitics, trade, culture, and the potential for countries to create mutual benefits. These interconnected factors have been emphasized in studies on nurse migration [5, 18–21]. Other factors include distress and displacement caused by conflict and insecurity, persecution, environmental degradation and limited opportunities [17, 18]. Moreover, the processes used in the international recruitment of African migrant nurses are varied and not uniformly regulated across international borders [9, 14, 22–29]. Such conditions can leave African migrant nurses vulnerable to exploitation and trafficking. Although evidence indicates a rise in the migration of African nurses, there is limited understanding of the recruitment strategies used to employ them. To develop effective interventions that safeguard African migrant nurses and address the challenges posed by nursing shortage, it is crucial to assess the current state of evidence surrounding African nurse migration.

## Objectives

The objectives of this review include: 1) To map and synthesize literature on the decisions surrounding African nurse migration; and 2) To summarize migration destinations and recruitment processes of African nurses.

## Methods

This review was guided by the Joanna Briggs Institute (JBI) updated methodological guidance for conducting scoping reviews [30], and findings were reported in accordance with Reporting Items for Systematic Reviews extension for Scoping Reviews (PRISMA-ScR).

### Data sources and searches

Migration studies is multidisciplinary and draws on diverse literature. The search strategy was guided by Pollock et al.’s [31] framework, and designed in consultation with a research librarian using text words from titles and abstracts from identified articles including, keywords designating nurse migrants (“*RN”* OR “*internationally-educated" OR “internationally educated”* OR *“overseas-trained”* OR “*overseas trained” OR* “*foreign-educated”* OR “*foreign educated”* OR “*foreign trained”* OR “*foreign-trained”* OR “*overseas-educated”* OR “*overseas educate*d” OR “*international professional”*) in combination with the terms designating migration (“emigrat*” OR “immigrat*” OR *“Mobility”* OR *“Moving”* OR “relocate*” OR “migrat*” OR “travel*”) and context (i.e., Africa*)*.* The final search spanned 16 databases to identify both peer-reviewed and grey literature including Cumulative Index to Nursing and Allied Health Literature (CINAHL), Medline, Web of Science, Scopus, Embase, EBSCO, Africa Bibliography, psych Info, databases of World Health Organization (WHO) [Africa Index Medicus], International Centre for Nurse Migration (ICNM), International Council for Nurses (ICN) and International Organization for Migration (IOM), International Labour Organization (ILO) and ProQuest Dissertations. The search was conducted in May 2023.

### Study selection

**Fig. 2:**
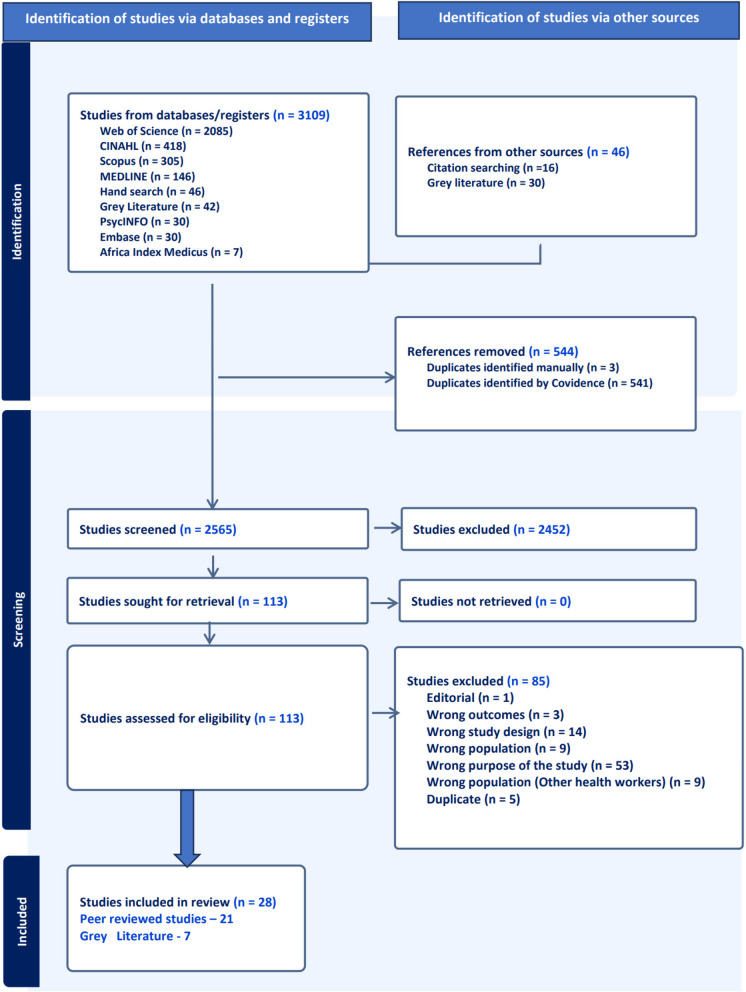
PRISMA flow chart showing the identification of studies

Studies were included if they reported empirical evidence on the experiences or factors influencing the migration decisions of participants who trained as nurses, had work experience in Africa, and had migrated to other countries for nursing work. Studies were also included if they were published in English from the year 2000 onward. This timeframe was chosen because the migration of nurses from the African continent gained momentum in the early 2000s [19, 32–35]. Studies were excluded if they reported exclusively on the intention of nurses or student nurses to migrate. We excluded literature reviews and conceptual papers. Citations retrieved from the database searches were imported to Zotero and then uploaded to Covidence, where duplicate results removed. Two independent reviewers screened titles and abstracts, followed by the full texts. Any disagreements between reviewers were resolved through consensus. Figure 2 shows the PRISMA diagram outlining the data screening process.

### Data extraction

After screening, we designed and integrated the data extraction tool into Covidence, with the first author and co-reviewer independently performing extraction. We extracted characteristics of included studies, such as author, study titles, publication year, data collection locations and methods. We then extracted findings relevant to the review’s objectives into Excel. Table 1 presents the characteristics of included studies.

**Table 1:** Detailed characteristics and findings of included studies

Reference	Author/Year	Country where data were collected	Push	Pull	Recruitment strategies	Preferred destination	Method	Design	Sampling Technique	Gender	Peer reviewed
[36]	Aboderin, 2007	U.K (Destination)	*	*			Qualitative	Exploratory qualitative	Purposive	Majority (Female)	Yes
[37]	Asampong, 2013	Ghana (Source)	*	*		OECD area	Qualitative	Exploratory case-study approach	Purposive	Majority (Female)	Yes
[34]	Bidwell 2014	U.K (Destination)	*	*		U.K	Qualitative	Descriptive Exploratory	Purposive	Majority (Female)	Yes
[35]	Buchan 2006	U.K (Destination)	*	*		U.K	Quantitative	Cross sectional	Convenience (online)	Majority (Female)	Yes
[38]	deVries 2016	Africa (Source)	*	*		U.K, U. S	Quantitative	Logistic regression	Purposive	Not specified	Yes
[39]	Organization for Economic Cooperation and Development, 2004	South Africa/OECD (Source)	*	*		OECD countries	N/A	N/A	N/A	Not specified	No
[40]	Hardill & McDonald 2000	U.K (Destination)	*	*		U.K	Qualitative	Case study	Purposive	Majority (Female)	Yes
[41]	Hashish & Ashour, 2020	Egypt (Source)	*	*		Middle East	Mixed methods	Descriptive Exploratory/cross sectional (concurrent triangulation)	Random/Purposive	Majority (Female)	Yes
[42]	International Council of Nurses, 2022	Global (Destination)	*	*	*	OECD countries	N/A	N/A	N/A	Not specified	No
[43]	Iyiola & Armstrong, 2018	Africa (Source)	*	*		OECD countries	N/A	N/A	N/A	Not specified	Yes
[44]	Jirovsky et al, 2015	Austria (Destination)	*	*			Qualitative	Descriptive exploratory	Purposive	Majority (Female)	Yes
[45]	Kingma, 2001	Switzerland/ICN (Destination)	*	*		North America, Western Europe, Asia, Africa, The Caribbean	N/A	N/A	N/A	Not specified	Yes
[46]	Labonte, 2015	South Africa (Source)	*	*			Mixed methods	mixed methods Comparative approach	Purposive	Majority (Female)	Yes
[13]	Lawal et al, 2022	Nigeria (Source)	*	*		U.K, U.S, Canada, Australia, Saudi Arabia	N/A	N/A	N/A	Not specified	Yes
[47]	Likupe 2013	U.K (Destination)	*	*		U. K	Qualitative	Descriptive exploratory	Purposive	Majority (Female)	Yes
[48]	Lowe & Chen, 2016	West Africa/WAHO (Source)	*	*			Quantitative	Cross sectional	Purposive	Majority (Male)	Yes
[46]	Swinburn, 2008	U.K (Destination)	*	*			N/A	N/A	N/A	Not specified	No
[49]	Oosthuizen et al, 2005	South Africa (Source)			*	Middle East, OECD areas	Mixed methods	Cross sectional	Purposive	Not specified	Yes
[50]	Poppe et al, 2014	Austria and Belgium (Destination)	*	*			Qualitative	Descriptive exploratory	Purposive	Majority (Female)	Yes
[51]	Pretorius, 2018	South Africa (Source)	*	*	*		Qualitative	Descriptive (Husserlian) Phenomenology	Purposive	Majority (Female)	Yes
[52]	Simplice, 2015	Africa (Source)	*	*			Quantitative	Quintile regression	Purposive	Not specified	Yes
[53]	Slote, 2011	Ghana (Source)	*	*			N/A	N/A	N/A	Not specified	No
[54]	Swinburne, 2007	U.K (Destination)	*	*			N/A	N/A	N/A	Not specified	No
[55]	Troy et al, 2007	Ireland (Destination)	*	*	*		Qualitative	Phenomenological	Purposive	Majority (Female)	Yes
[56]	Thupayagale- Tshweneagae 2007	Botswana (Source)	*	*		New Zealand, U.K, U.S, Australia	N/A	N/A	N/A	Not specified	Yes
[57]	Vujicic et al, 2004	Switzerland/WHO (Destination)	*	*		U.K, Canada, U.S	Quantitative	Correlational	Purposive	Not specified	Yes
[58]	WHO, 2020	Africa (Source)	*	*			N/A	N/A	N/A	Not specified	No
[36]	WHO, 2022	Africa (Source)	*	*			N/A	N/A	N/A	Not specified	No

* Indicates findings in the study, *N/A* Not Applicable

*Source – This indicates that data was obtained from a source country

*Destination – This indicates that data was obtained from destination country

### Analysis and presentation of results

After data extraction, we synthesized evidence from the included studies and identified high level themes. For more complex themes, we further identified sub-themes to reduce complexity and also provide a multidimensional description [45]. All data were presented in a narrative summary.

### Findings

We included 28 studies, published from 2000 onwards, comprising twenty-one peer-reviewed studies and seven reports from the grey literature. More than half (*n* = 53.6%, *n* = 15) of the included studies were conducted in source countries, while 46.4% (*n* = 13) were conducted in destination countries, including 25% (*n* = 7) from the United Kingdom. Among the twenty-eight studies, nine (*n* = 9, 32.1%) used qualitative methods, six employed descriptive exploratory designs (*n* = 6, 21.4%), and three of the included studies were mixed methods studies (*n* = 3, 10.7%). The majority (*n* = 16, 57.1%) utilized a purposive sampling technique. In nine studies (*n* = 16, 57.1%) participants recruited for data collection in nine out of the 28 included studies (*n* = 9, 32.1%), participants typically fit a specific profile: they were female, married, over 30 years old, and had at least one dependent child. In terms of gender, fifteen out of the 28 included studies did not specify the gender of participants. Studies were conducted across Africa, Europe and by international organizations with nearly a third of the included studies (*n*= 8, 29%) taking place in the United Kingdom and Ireland, followed by four in Southern Africa (*n* = 4, 17.9%).

The synthesis identified three main themes: factors influencing international African nurse migration, factors affecting African nurses’ destination choices, and recruitment strategies for the employment of African nurses.

### Theme: factors influencing international African nurse migration

The factors influencing African nurse migration include career dynamics and job sustainability, economic challenges and income disparities, personal and social factors, and safety and security challenges. These findings suggest that the factors driving nurses’ migration are highly multidimensional.

#### Subtheme: career dynamics and job sustainability

Twenty studies identified career dynamics [37, 40, 41, 47, 48, 50, 54, 56–59] and job sustainability [36, 38, 41–44, 51–53, 55] as key factors in African nurse migration decisions. For this synthesis, career dynamics refers to learning opportunities within the context of nursing employment to enhance skills, knowledge and career prospects. Job sustainability, on the other hand, describes the ability of nurses to remain satisfactorily employed while having access to opportunities to upgrade and grow their careers. The World Health Organization (WHO) [41] in its *State of the World Nursing Report, higlighted* that nurses aspire to professional growth and development and are motivated to seek opportunities to practice to the full scope of their skills and knowledge. This can include clinical skills training and general nursing education [41]. Iyiola and Armstrong [36] and Asampong et al. [44] assert that lack of or limited opportunities for clinical skills training and education negatively impacts nurses' professional skills, education and growth. Studies in this review identified challenges such as their regular availability of in-service training programs and a lack of funding for nurses to undergo professional training. These findings were supported by mixed methods studies in Ghana [44] and Egypt [53], as well as an interview of Nigerian nurses in the United Kingdom [46]. Consequently, many nurses migrate to seek opportunities for education and career growth [56, 58]. In a cross sectional survey of African nurse migrants working in London, Buchan et al. [48] reported that participants sought opportunities abroad to pursue further education, upgrade their skills, gain experience, and to work in environments that allow them the opportunity to practise to the full scope of their abilities [40, 41, 59]. African nurses also face limited job and career growth opportunities, which hinder their career prospects and motivate them to migrate out of their home countries [36, 39, 48]. Simplice [38] reported that some nurses experienced job stagnation without promotion or were at risk of losing their jobs due to financial constraints confronting African health systems. Poor job conditions and job insecurity contribute to high rate of unemployment in Africa, thus further limiting nurses’ career prospects [48, 51]. Joblessness and job insecurity therefore influence nurses to migrate in search of better opportunities and improved their career prospects.

#### Sub-theme: economic challenges and income disparities

Economic challenges and income disparities significantly impact African nurses [39, 41]. Nurses’ ability to earn a decent living is impaired by the general economic decline in their home countries, prompting many to migrate to destinations offering competitive wages and remuneration [41, 50, 59].

Nineteen studies (60.7%) stressed the role of economic challenges and income disparities in motivating the migration of African nurses to other countries in pursuit of better compensation and improved standards of living [36, 38–40, 42–44, 46, 48–50, 53, 54, 57, 59–62]. Dating back to the 1980s, Aboderin [46] in an exploratory qualitative study, highlighted the migration of Nigerian nurses in response to Nigeria's economic challenges, which led to declining incomes due to inflation. Recent evidence suggest that similar conditions persist in many African economies [38], further impacting the ability of health systems to adequately compensate nurses, including through salaries, pensions and other benefits. Consequently, African nurses have migrated to destinations with better work benefits [43, 49, 57, 61]. For example, a mixed methods study among Egyptian nurses found that low-income levels influenced their decision to migrate to more lucrative destinations [53]. Similar reasons were cited by Ghanaian nurse-returnees in a case study [44] and in a survey of African nurse migrants in the UK [48]. Zambian nurses, compelled to migrate due to income challenges, expressed a desire to remain in their home country if their remuneration were sufficient to meet their basic needs and improve their quality of life [57]. These findings suggest that economic decline leads to resource-constrained health delivery systems, low wages, and salaries, making it difficult for nurses to meet their basic needs and work comfortably. Moreover, Iyiola and Armstrong [36], in a peer reviewed article on open and free migration of nurses in Sub-Saharan Africa, found that low-income levels and economic challenges remain key drivers of African nurses’ migration. In a correlational study on wage differentials and nurse migration, Vujicic et al. [40] found that income differences between nurses in Africa and those in destination countries, such as those in the Organization for Economic Cooperation and Development (OECD) region, are so significant that improvements in African nurses' may not substantially reduce migration rates. 

#### Subtheme: personal and social factors influencing nurse migration

Social factors and the pursuit of personal ambitions influenced the decision of some nurses to migrate to other destinations. Thirteen studies emphasized personal and social factors that motivated African nurses to migrate [33, 36, 38, 42, 46, 48, 52–54, 58, 59, 61, 63]. Family and personal networks of nurses living in destination countries could play a significant role in driving African nurse migration. Through marriage, family ties and personal networks, nurses migrate from the continent [48, 52, 59]. Also, personal networks facilitate migration by the provision of vital information and assisting in the easy integration of African nurses into their destination environments [58]. This indicates that family-related and social networks are significant drivers of nurse migration and play a multifaceted role in re-uniting families. Family reunification and personal networks thus drive mobility and create new opportunities for African nurses in destination countries.

Moreover, the loss of social recognition and the declining public image of nurses in some African countries have influenced their decision to migrate. In reporting about Nigerian nurse migrants in the UK, Aboderin in a qualitative study [46] found that the strained relationship between doctors and nurses resulted from changes in the nature of nursing care in Nigeria. These changes shifted nursing from a “vocation” to a profession, emphasizing a more scientific approach to patient care, professionalism, and autonomy. Influenced by developments in the nursing profession in U.K and United States, nurses in Nigeria became more assertive and increasingly protested against their non-recognition of nurses as professional equals by doctors who often viewed nurses as merely carers. Nurses also resisted the patronizing attitudes of some doctors [46]. Consequently, the public no longer perceived nurses as devoted carers but as arrogant and condescending towards patients [46]. For instance, Hashish and Ashour [53] reported in a mixed methods study that Egyptian nurses felt unappreciated at work and by the public. The response of a Nigerian participant in a qualitative study by Aboderin [46] vividly illustrated the perceived struggle for recognition by African nurses.

*“There has been a paradigm shift. Caring is…still the basis of nursing. But professionalism, autonomy…have been given emphasis. And… assertiveness…The philosophy is: you are a professional…you… interact with a patient, analyse the problem, come up with the diagnosis and design a care plan within the jurisdiction of nursing...*” (Nigerian Nurse)

Furthermore, some African nurses seeking international experience are more likely to migrate, even when conditions of work are favorable. Some nurses are attracted by the appeal of multiculturalism, diversity, international experience and curiosity [52, 53]. Others seek destinations offering diverse cultural experience, and a lifestyle free from scrutiny [38, 54]. Similarly, some nurses report being drawn by world class health facilities, desiring the experience and privilege of working in such environment [52]. For instance, a South African nurse in her early 40s submitted the below in a study by Hardill and McDonald [52] in U.K.

*“……… I have always loved to visit First World hospitals. To compare things and get more knowledge and what is happening in Nursing”.* (South African nurse)

#### Subtheme: safety and security challenges

Participants in eight studies cited safety concerns in the workplace as a key factor in their decision to migrate [43, 47, 51, 52, 57, 58, 63, 64]. In this review, safety refers to the provision of safe workplace environment free from high risks of injury. High workloads, long working hours, and the use of obsolete equipment, create hazardous working conditions for nurses in many African countries, and thus influencing them to migrate in search of safer and more acceptable levels of work [51, 57]. In an article on the migration of nurses in Botswana, Thupayagale-Tshweneagae [58] found that declining number of nurses in the Botswana healthcare system led to longer working hours and, ultimately, high stress levels among nurses in the public health delivery sector. The risks of exposure to different biological and physical hazards in the workplace are exacerbated by heavy workloads. This is reiterated in a special article by Lawal et al. [43] on brain drain in Nigeria during the COVID-19 pandemic, which showed how lack of equipment increased the exposure of Nigerian nurses to infectious disease such as COVID-19, resulting in many fatalities. Also, tension among nurses in the workplace can create a hostile atmosphere which fosters a sense of insecurity among nurses. In a qualitative interview of South African migrant nurses working in the UK, participants reported bullying by colleagues as a driving factor in their migration to the UK [52]. Thus, issues surrounding safety in the workplace play a significant role in influencing the decision of nurses in Africa to migrate.

Security challenges in the form of violence and political instability, have also created distressing situations that forces nurses in Africa to migrate. Violent crimes, political unrest, gender-based violence, and persecution of intellectuals in parts of the continent have compelled nurses to seek safer destinations [36, 38, 42, 53, 59]. Jirovsky [42] and Poppe et al. [59] in separate qualitative interviews, highlighted the migration of Congolese nurses to Belgium and Austria as they fled violence to practice their profession in safer destinations. The factors influencing the migration of African nurses are multidimensional, reflecting the complex challenges confronting Africa’s health delivery systems, ranging from systemic to personal factors.

### Theme: recruitment strategies in the employment of African nurses

Recruitment agencies utilized multiple and complex strategies to recruit nurses from Africa [52, 57, 64], highlighting their central role in the regulation of nurse migration and advancing UHC. Over two decades ago, McDonald and Hardill [52] and Swinburne [57] reported in separate qualitative studies and, Oosthuizen et al. [64], in a mixed methods study, reported that recruitment agencies based in destination countries such as United Kingdom and the United States recruited nurses directly from South Africa, Zambia and other African countries. These agencies used online advertisements, as well as ads in magazines and medical journals. Recruitment agencies based in destination countries also collaborated with local agencies in African countries to recruit nurses. Similarly, local recruitment agencies also facilitated direct recruitment for destinations such as Saudi Arabia [54]. Again, through international inter-agency collaboration, recruitment agencies advertised in medical journals and near hospitals where nurses were employed [54]. Oosthuizen et al. [54], in a mixed methods study, found that nurses in South Africa inquired with local agencies about potential recruitment opportunities to preferred destinations.

### Theme: factors influencing African nurses’ destinations choices

Findings from several studies [44, 47, 49, 54] highlight the factors influencing the choice of migration destinations of African nurse migrants. De Vries et al. [49] argued that cultural and linguistic factors are vital in determining the choice of migration destinations. They found that Portuguese-speaking Mozambican nurses often migrated to either Brazil or Portugal, while Arab-speaking Egyptian nurses favored the Middle East [49]. Anglophone African nurses from Nigeria, [46], Ghana [44] and South Africa [52] preferred the United Kingdom and other English-speaking countries due to historical colonial relationships, language similarities, visa accessibility and easy integration into the healthcare system of destination countries [44, 46, 52]. Cultural congruence also influence the choice of destinations, as evidenced by a mixed methods study of Egyptian nurses [53]. For example, Muslim Egyptian nurses selected Middle Eastern destinations such as Saudi Arabia to perform Umrah and Hajj.

## Discussion

This review synthesizes evidence on African nurse migration, exploring the factors that influence their migration, shape their choice of destinations, and drive recruitment strategies. It higlights the central roles of economic factors, job security, and career development in shaping the decisions and migration pathways of African nurses.

### Career dynamics and job sustainability

As a competency-based occupation, nursing requires continuous skills improvement and professional development to advance competence in practice and professionalism. These efforts are required for nurses to remain professionally competent, enhance their chances for retaining employment, gaining promotions, or seeking new positions. Thus, the pursuit of career development and job security opportunities serves as a powerful motivator for migration. Consequently, nurses in African countries who lack access to opportunities for continuous professional development and job security are more likely migrate in search of better opportunities to develop professionally, and improve their employment prospects, particularly for unemployed nurses. The findings of Hardill and Macdonald [52], Likupe [54] and Pretorius [60] are corroborated by the World Health Organization's report on optimizing and accelerating investments in nursing and midwifery for resilient health systems in Africa [55], which contend that promoting the professional development and competence of Africa's nursing workforce can play a significant role in retaining nurses. Therefore, it can be inferred that improved access to continuous professional development and the creation of more job opportunities could promote the retention of nurses in the African continent.

### Economic challenges and income disparities

Economic challenges in many LMICs, including those in Africa, continue to create difficult living conditions for their populations including nurses. These challenges lead to declining disposable incomes and increase the likelihood of emigration, thus, further impeding efforts at scaling up and optimizing nursing workforces across the continent [38]. Economic issues such as high taxation, inflation, low wages and inadequate pensions place disproportionate financial burdens on nurses [46]. Consequently, these demanding economic conditions raise the cost of living and influence the decision of nurses to migrate to seek better economic conditions and higher income. The International Organization for Migration [17] and the International labor Organization [65] emphasize that economic factors continue to play an essential role in driving labor migration, including that of nurses. In an analysis of secondary data on the challenges and issues related to the migration of nurses from Sub-Saharan Africa, Dovlo [32], a health systems consultant, found that nurses and other health care workers migrate out of the continent due to structural economic circumstances that create high costs of living and make life particularly unbearable for nurses. These long-standing economic challenges in Africa are often linked to credit programs, such as the economic adjustment program rolled out by the World Bank and the International Monetary Fund, which imposed austerity measures that limited recruitment and job opportunities for African nurses and other public sector workers [66]. This synthesis builds on previous evidence regarding the impact of economic factors on the labor migration of African health workers [5, 21] and provides a clearer understanding of how structural economic factors in influence nurse migration from the continent. Improving economic conditions in the continent could help reduce nurse migration, scale up and optimize nursing workforces, and improve access to nursing care.

### Personal and social factors

This synthesis establishes that the desire for social recognition, family ties, and other social networks drive African nurse migration [48, 52, 59]. Migration acts as a bridge to connect family members and personal networks, as well as a means for nurses to seek social recognition. These findings align with a WHO [35] synthesis report on health professionals’ migration in six African countries (Ghana, Senegal, Cameroon, South Africa, Uganda and Zimbabwe) and a qualitative study by Ryan [67] on family-led migration among Irish nurses, which highlighted that family-related factors are significant drivers of nurse migration and play a major role in reuniting families. Deeply embedded in a collectivist cultural framework of many Africa societies is an emphasis on the desire to forge a sense of community and meaning [68]. This is reflected in the obligation to show loyalty to family and group members. As a result, African nurses in desirable destination countries often support and influence family members and other social networks, including unemployed professional nurses, thereby and driving further migration. Again, as the nursing profession in Africa evolves, the growing assertiveness of nurses and their desire for recognition as professionals - rather than mere caregivers - can frustrate those who lack professional and public recognition, accelerating their migration for social and professional validation. From this review, it can be argued that pursing nursing as a profession can improve chances of migration, reuniting with family members, social networks and the opportunity to explore new places. 

### Safety and security challenges

Nurses also prioritize safe, modern, and fair working conditions when making migration decisions. This evidence synthesis highlights that the quests for safety and wellbeing has been a catalyst for migration [49, 53, 58]. In addition, high workload and long working hours lead to stress and eventual burnout, while, broken equipment and inadequate resources increase the risks of nurses’ exposure to biological and physical hazards, compromising their health and well-being. This is evident in the case of nurses in the Philippines, where, Alibudbud [69], in a commentary on addressing stress and nursing shortages, emphasized that burnout and stress lead to migration, resignations, or career changes. Similarly, a secondary data analysis by Davis [70] from the United States, observed that one-third of nurses planned to either migrate or quit their jobs due to stress and burnout by their working environment. This issue was pronounced during the COVID-19 pandemic where in the WHO's *State of the World’s Nursing* report [41], which noted that healthcare workers worldwide, including nurses, faced various forms of work-related stress, including abuse from patients and their families. Beyond creating unsafe working conditions, high stress levels can lead to increased absenteeism, further exacerbating unfair workload and shortages among nurses. Consequently, stress and burnout drive migration, creating nursing shortages, that in turn heighten workplace stress and burnout, perpetuating a vicious cycle.

Moreover, insecurity and violence cause displacement and distress migration among nurses in the continent [36, 38, 42, 53, 59]. The Organization for Economic Cooperation and Development (OECD) [63] reported South African nurses sought safety from violent neighborhoods, while Congolese nurses fled political violence and instability to Belgium and Austria, as documented in a qualitative investigation on the motivations for African nurse migration [61]. These findings are supported by the WHO's *state of the world’s nursing report * [41], which reiterates the role of violence in driving nurse migration and undermining the delivery of quality healthcare. This implies that nurses facing violence and political instability may flee to safety, even to countries, not traditionally known for recruiting nurses, such as Austria. Violence and insecurity exacerbate nurse migration, and causes declines in the nursing work force in source countries, especially in volatile regions of the continent.

### Recruitment strategies

Recruitment plays an essential role in regulating the migration of African nurses. The plethora of strategies revealed in this synthesis include international collaboration between local and destination agencies; partnerships between destination hospitals and local agencies; direct destination recruitment by destination hospitals through social media and medical journals; and unsolicited applications by nurses through local agencies for potential recruitment [52, 57, 64]. These recruitment strategies suggest the diverse and often unregulated ways in which nurses are recruited. The findings of this review vary significantly from the strategies described by Smith et al. [25] in a qualitative study in the United States, where hospital champions acting as recruitment ambassadors on social media were found to be a key recruitment method. This approach also contrasts with findings from another U.S-based qualitative study [24], which emphasized the importance of integrating preceptorship into recruitment to promote retention of nurses in hospitals, and from a phenomenological study in Sweden [29], where participants acknowledged the role of partnerships between health facilities and schools of nursing in recruiting new graduates. These variations in recruitment strategies for African nurses, compared to other regions, indicates the potential for adopting additional strategies for Africa. As recruitment strategies become more diverse, the adoption of more advanced strategies in the age of increased access to internet in the African continent can exacerbate African nurse migration. However, this raises numerous concerns about the unregulated nature of recruitment processes in the continent. There is a need for a streamlined framework that governs recruitment standards in Africa. Although not legally binding, calls for greater recognition and enforcement of the World Health Organization’s Global Code of Practice on the International Recruitment of Health Personnel [71] would help monitor and standardize regulations globally.

### Destinations of African nurses

This review found that nurses migrate to countries both within and outside Africa, with their choice of destination being diverse and impacted by multiple factors. In the past, Nurses from countries such as Ghana and Zimbabwe primarily moved to South Africa, but this trend has recently shifted towards countries in the OECD area due to higher spending power and better conditions of work [52, 58, 63]. The migration patterns and destination choices of African nurses to the global north highlight the impact of past colonial relationships in facilitating labor migration [6, 54, 63]. Educational and linguistic similarities between source and destination countries have enabled the migration of nurses from Anglophone African countries, such as Ghana and Nigeria to English-speaking destination countries like the United Kingdom, as well as the or migration of Portuguese-speaking nurses from Mozambique to Portugal and Brazil. The broader literature on nurse migration and the global healthcare economy [1, 19, 20] corroborates this review’s findings on the migration of nurses from African countries such as Ghana, South Africa and Zimbabwe to the United Kingdom and Ireland, emphasizing the role of colonial and cultural ties in facilitating African nurse migrants' destination choices. Historical relations continue to influence and facilitate the choice of destinations of African nurse migrants.

### Implications for policy, practice and research

Nurses governments in Africa and international organizations should prioritize raising nurses' wages to realistic levels and creating social safety systems to support nurses facing economic hardships. Policies could include scholarship programs for nurses' children, subsidies for housing, healthcare support to improve their quality of life. Furthermore, governments must implement policies aimed at reducing violence, resolving conflicts, and promoting political stability to create safer working environments for nurses and other health care professionals. Moreover, clear regulations and standards for nurse recruitment agencies to protect the rights, welfare, and interests of nurses seeking employment abroad. This includes enforcing ethical recruitment practices and monitoring compliance with international guidelines, such as the WHO's *Global Code of Practice on the International Recruitment of Health Personnel. *Again, it is pertinent for nursing institutions and healthcare organizations should invest in continuous learning and professional development opportunities to enhance nurses’ skills, knowledge, and career prospects. Addressing the lack of regular in-service training is crucial for improving nurses' competencies and promoting retention. As African nurse migration continue to rise, further studies are needed to examine the migration and transition experiences of African nurses, including the challenges they face and the support systems that best facilitate their their adaptation in destination countries.

### Limitations of the study

This review included only literature published in English from the year 2000 onward, which may exclude literature published in languages or before 2000. Future reviews should consider a wider range of publication years. Again, the review focused heavily on data from the United Kingdom and a few other destinations, which may not fully represent the global situation. Future reviews should include diverse geographical data to provide a more comprehensive perspective. 

## Conclusions

Low income, poor economic growth, and inadequate investment in African health systems significantly drive the emigration of African nurses, complicating efforts to achieve universal health coverage. Recruitment strategies for nurse from African are often unregulated, which can lead to exploitation and human trafficking. As African nurse migration continues to rise, further studies is needed to examine their migration and transition experiences, as well as support systems available in destination countries. Finally, improving workforce policies to meet the evolving needs of nurses is vital for retaining nurses in Africa.

## Data Availability

The data used for this current study are available on request from the corresponding author.
